# Establishment of a Rapid Lesion-Controllable Retinal Degeneration Monkey Model for Preclinical Stem Cell Therapy

**DOI:** 10.3390/cells9112468

**Published:** 2020-11-13

**Authors:** Guanjie Gao, Liwen He, Shengxu Liu, Dandan Zheng, Xiaojing Song, Wenxin Zhang, Minzhong Yu, Guangwei Luo, Xiufeng Zhong

**Affiliations:** 1 State Key Laboratory of Ophthalmology, Zhongshan Ophthalmic Center, Sun Yat-Sen University, Guangzhou 510060, China; gaogj5@mail2.sysu.edu.cn (G.G.); heliwen@mail.sysu.edu.cn (L.H.); liushx26@mail2.sysu.edu.cn (S.L.); zhengdandan@gzzoc.com (D.Z.); songxiaojing@gzzoc.com (X.S.); zhangwenxin@gzzoc.com (W.Z.); luoguangwei@gzzoc.com (G.L.); 2 Electrophysiology Laboratory, Department of Ophthalmology, University Hospitals, Case Western Reserve University, Cleveland, OH 44101, USA; minzhong.yu@uhhospitals.org

**Keywords:** animal model, monkey, retinal degeneration, sodium nitroprusside, stem cell therapy

## Abstract

Background: Retinal degenerative disorders (RDs) are the main cause of blindness without curable treatment. Our previous studies have demonstrated that human-induced pluripotent stem cells can differentiate into retinal organoids with all subtypes of retina, which provides huge promise for treating these diseases. Before these methods can be realized, RD animal models are required to evaluate the safety and efficacy of stem cell therapy and to develop the surgical tools and procedures for cell transplantation in patients. This study involved the development of a monkey model of RD with controllable lesion sites, which can be rapidly prepared for the study of preclinical stem cell therapy among other applications. Methods: Sodium nitroprusside (SNP) in three doses was delivered into the monkey eye by subretinal injection (SI), and normal saline was applied as control. Structural and functional changes of the retinas were evaluated via multimodal imaging techniques and multifocal electroretinography (mfERG) before and after the treatment. Histological examination was performed to identify the target layer of the affected retina. The health status of monkeys was monitored during the experiment. Results: Well-defined lesions with various degrees of retinal degeneration were induced at the posterior pole of retina as early as 7 days after SNP SI. The damage of SNP was dose dependent. In general, 0.05 mM SNP caused mild structural changes in the retina; 0.1 mM SNP led to the loss of outer retinal layers, including the outer plexiform layer (OPL), outer nuclear layer (ONL), and retinal pigment epithelium (RPE); while 0.2 mM SNP impacted the entire layer of the retina and choroid. MfERG showed reduced amplitude in the damaged region. The structural and functional damages were not recovered at 7-month follow-up. Conclusion: A rapidly induced lesion site-controllable retinal degeneration monkey model was established by the subretinal administration of SNP, of which the optimal dose is 0.1 mM. This monkey model mimics the histological changes of advanced RDs and provides a valuable platform for preclinical assessment of stem cell therapy for RDs.

## 1. Introduction

Retinal degenerative disorders (RDs) with different pathogeneses, such as age-related macular degeneration (AMD) and retinitis pigmentosa (RP), may cause the dysfunction or degeneration of retinal pigment epithelium (RPE) and/or photoreceptors and finally cause blindness [1,2]. AMD currently affects about 180 million people worldwide, and the prevalence tends to increase as the older population increases. Publications indicate that medication or gene therapy might be effective for the early stage of RDs with remaining photoreceptors [3,4,5]. There is no cure yet for middle- to end-stage RDs in which all photoreceptors and RPE are lost. Retinal cell transplantation has been regarded as a potential treatment to replace the lost cells and to restore their structures and functions as well [6,7,8]. With the rapid development of human pluripotent stem cell (hPSC) technology and successful reproduction of retinal cells and tissues with hPSCs [9,10,11,12,13,14], the bottleneck of retinal grafts, shortage of the cell or tissue donors, has been largely solved. Therefore, stem cell therapy holds huge promise for restoring the vision of RD patients. 

RD animal models are required to evaluate the safety and efficacy of retinal stem cell therapy and to develop the surgical tools and procedures for cell transplantation in patients [15]. Although RD-like animal models from several species, such as RD1 mice, RCS rats, and rabbits, have been widely used in the study of retinal stem cell transplantation, models of animals with eyeball size and structure closely similar to human counterparts would be preferred, especially for preclinical evaluation of cell therapy. Nonhuman primates like monkeys are one such animal, since they have not only the equivalent eyeball diameter but also the macula, a unique structure of a primate’s retina, responsible for detailed daytime vision and color vision [16]. Due to the high cost in purchasing and breeding these animals, establishment of an RD-like model in monkeys, which can be safely and rapidly prepared, mimicking the pathological features and being suitable for cell therapy study, is largely in demand. 

Retinal degenerative conditions in medium- and large-sized animals, such as cats, rabbits, pigs, and monkeys, have been induced by several different approaches, including laser or severe light exposure, genetic manipulation, and retinotoxic reagents [17,18,19,20,21,22,23,24,25]. However, some limitations exist in these models. For instance, laser or light exposure caused focal damage of the retina with small lesion size or inconsistent degree of damage [20,21], which is not suitable for cell transplantation study; transgenic RD monkey models are not yet available; intravenous injection of retinotoxic agents, such as sodium iodate, iodoacetic acid, and N-methyl-N-nitrosourea (MNU), led to systemic complications with high mortality, which is not applicable to larger animals, especially to monkeys due to the high cost of experiments; while intravitreal injection of the above agents caused uncontrollable or random distribution of retinal lesions [15,22,23,24], which might be suitable for medication or gene therapy of RD-like diseases but is not practical for the evaluation of retinal cell replacement therapy. The latter needs to directly and accurately deliver cells into the subretinal space of the lesion area to replace the lost cells or tissues. A new method of subretinal injection (SI) has been applied to deliver cobalt chloride to induce a focal RD-like model in monkeys for retinal sheet transplantation [19], but the characteristics of this type of focal RD-like model are not fully clear.

Sodium nitroprusside (SNP) is a clinically available drug that has been widely used to treat acute hypertension for decades. It can release nitric oxide (NO), a free radical gas with various physiological and pathophysiological roles in the retina [26]. Recently, several groups including us have reported that SNP is a safe agent and that intravitreal administration of SNP can lead to retinal degeneration in rats and rabbits [27,28,29]. Therefore, in this study, we subretinally delivered SNP into the posterior pole of retinas in monkeys. The dose–effect of SNP on retinal damage was evaluated in structural and functional levels by multi-modalities. An RD-like model with focal damage of the monkey retina was established and can be considered suitable for the preclinical assessment of stem cell therapy.

## 2. Materials and Methods

### 2.1. Animals

The study design and experimental protocols were approved by the Animal Ethics Committee of the Zhongshan Ophthalmic Center, Sun Yat-Sen University (no. 2016-012 and 2019-042). All experimental procedures involving animals adhered to the Association Research in Vision and Ophthalmology (ARVO) Statement for the Use of Animals in Ophthalmic and Vision Research. Fourteen healthy male cynomolgus monkeys (28 eyes) aged 1–4 years were used in this study and housed individually in stainless steel cages in an animal experimental room with environmental conditions of 16–26 °C room temperature, 40–70% humidity, and 12 h lighting (7 AM to 7 PM; illumination intensity >200 lux). The animals were generally fed 5% body weight/animal/day of pellet food, which was adjusted based on changes in the appetite and weight of the monkeys. Tap water from a feed-water nozzle was provided ad libitum to the animals.

### 2.2. Drug Delivery 

The SNP (Hongyuan, Dongguan, China) was dissolved in normal saline (NS) at three different doses (0.05, 0.1, and 0.2 mM) followed by filter sterilization and protection from light. The cynomolgus monkeys were anesthetized with intramuscular injection of 4–6 mg/kg (50 mg/mL) Zoletil 50 (Virbac, Carros, France) and topical application of 0.5% proparacaine hydrochloride eyedrops (Ruinian Best, Nanjing, China). Several subjects (n = 5) were given addition intramuscular injections of 1–3 mg/kg xylazine hydrochloride (Huamu, Changchun, China) to deal with eye movements. The pupils were fully dilated (≥7 mm) using tropicamide phenylephrine eyedrops (Mydrin-P) (Santen, Osaka, Japan) before surgery. Under a surgical microscope (M844F20) (Leica, Wetzlar, Germany), the sclerotic incision at 10:00 was performed through the pars plana (3 mm behind the limbus) using a 23 G trocar (Alcon, Fort Worth, TX, USA). Then, 100 μL of SNP solution of different doses or NS were injected into the subretinal space at the posterior pole of the retina next to the fovea via a Nanofil-100 microsyringe (WPI, Shanghai, China) with a 34 G needle (NF34BL-2) (WPI, Shanghai, China). Twenty-eight eyes from 14 cynomolgus monkeys were randomly divided into 4 groups. Six eyes received 0.05 mM SNP, 14 eyes 0.1 mM SNP, 5 eyes 0.2 mM SNP, and 3 eyes NS. Health indicators, such as weight, food intake, and daily activity, were monitored during the experimental period.

### 2.3. In Vivo Observation and Evaluation

The morphological changes of the retina were observed with a binocular indirect ophthalmoscope (HEINE OMEGA 500) (HEINE Optotechnik GmbH & Co. KG, Munich, Germany) and photographed with a digital fundus camera (TRC-50DX) (Topcon, Tokyo, Japan). Spectral-domain optical coherence tomography (SD-OCT), BluePeak autofluorescence (BAF), fluorescein angiography (FA), and indocyanine green angiography (ICGA) were performed with a SPECTRALIS HRA+OCT (Heidelberg Engineering, Heidelberg, Germany). The cynomolgus monkeys were anesthetized as above before examination. All procedures were performed according to the manuals of equipments.

SD-OCT images were acquired in high-speed mode with a 30° × 25° horizontal line scan with 9 frames averaged in each B scan. The total retinal thickness from the internal limiting membrane (ILM) to Bruch’s membrane (BM) and the inner retinal thickness from the ILM to the inner nuclear layer (INL) in the injection area were measured using the mode “Thickness Profile” of the software “Heidelberg Eye Explorer”. In this mode, a circular range with a diameter of 6 mm was placed in the center of the injection area, then the software automatically calculated the average thickness.

BAF images were acquired using a confocal scanning laser ophthalmoscope (cSLO), which had a 488-nm excitation filter and a 500-nm barrier filter and was equipped with an internal fluorescent reference for the correction of variable laser power and differences in detector sensitivity.

FA and ICGA were performed 5 months after SNP injection according to the reported study [30]. The cSLO of the SPECTRALIS HRA+OCT had a 785-nm excitation filter and an 820-nm barrier filter for ICGA and a 488-nm excitation filter and a 500-nm barrier filter for FA. In each attempt, 0.1 mL/kg of 10% sodium fluorescein (Baiyunshan, Guangzhou, China) or 2.5 mg/kg of indocyanine green (ICG) (Ruidu, Dandong, China) was administered intravenously. FA was performed first and completed in 15 min. Next, ICGA was performed and completed in approximately 40 min. Videos of the first minute of FA and ICGA were recorded, and pictures of the angiograms were taken every 15 s after 1 min from the beginning.

### 2.4. Histological Evaluation

The monkeys were euthanized using a lethal dose of potassium chloride (35 mg/kg) injected into the cephalic vein 7 months after SNP treatment. Right after death, both eyes were enucleated and immersed in a mixture of 10% neutral-buffered formalin and embedded in paraffin. Then, 5-μm-thick sections of the injected area were cut and stained with hematoxylin and eosin (H&E). Immunohistochemistry staining with the first antibody recoverin (1:500) (EMD Millipore, Temecula, CA, USA) was also performed. The slides were examined to detect pathological changes in the retina using a light microscope (Axio Scan Z1) (Zessi, Jena, Germany). 

### 2.5. Functional Evaluation

The functional changes of SNP-treated retinal lesions were evaluated with a noninvasive RETImap multifocal electroretinogram system (Roland Consult, Brandenburg, Germany) according to the International Society of Visual Clinical Electrophysiology (ISCEV) standards [31]. The pupils were fully dilated (≥7 mm) using tropicamide phenylephrine eyedrops. The corneas were anesthetized using 0.5% proparacaine hydrochloride eyedrops. A gold foil annular corneal active electrode was placed onto an anesthetized cornea, a ground electrode was placed onto the skin on the forehead, and a reference electrode was placed 1 cm outside the orbital rim. During the recording, the fundus was monitored with an SLO to make sure the stimulus pattern was consistently positioned on the injection area of retina. The stimulated region consisted of 37 unscaled hexagons with a total diameter of 25°. After each stimulus cycle, a fundus photograph from the SLO was taken to record the corresponding fundus area. The multifocal electroretinography (mfERG) tests were repeated for 6 cycles. The right eye and left eye were tested individually. After completion of the test, tobramycin eyedrops were administered to each eye. 

### 2.6. Statistical Analysis

Results are presented as mean ± standard deviation (SD). Data were analyzed using t-test. Statistical analysis was performed using GraphPad Prism software (GraphPad Software, San Diego, CA, USA). The criterion for statistical significance was *p* < 0.05.

## 3. Results

### 3.1. Subretinal Administration of SNP-Induced Controllable Focal Retinal Degeneration in Cynomolgus Monkeys 

**Figure 1:**
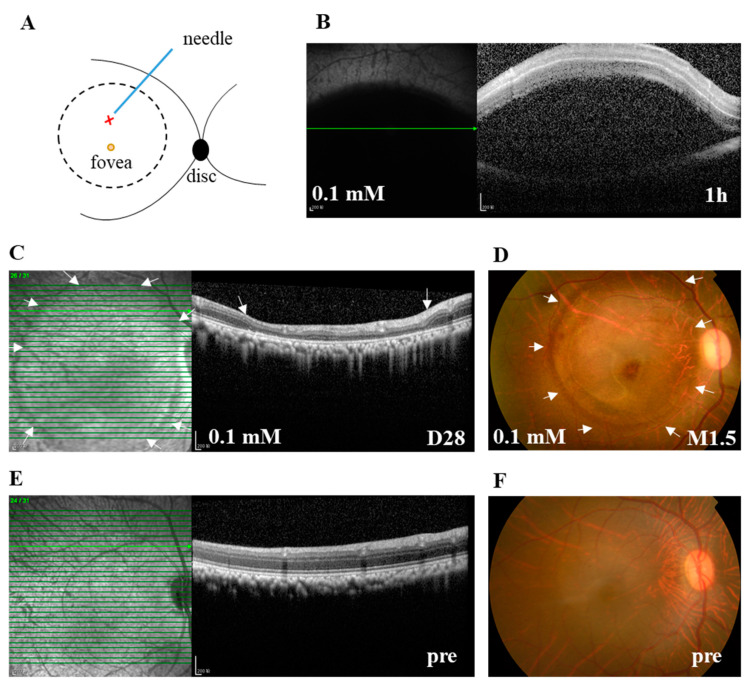
The subretinal injection (SI) of sodium nitroprusside (SNP) caused focal retinal lesion in monkeys. (**A**) Illustration depicts the SI site at the posterior pole of the retina. The retinotomy site by a needle is indicated with X. The bleb area caused by SI is indicated with a circle. (**B**) A typical spectral-domain optical coherence tomography (SD-OCT) image confirming the formation of subretinal bleb 1 h after SI, which indicated the successful delivery of SNP solution. (**C**) Example of SD-OCT images showing the focal retinal degeneration induced by 0.1 mM SNP SI on day 28 (D28) after the SI. (**D**) Fundus photograph also showing the local lesion surrounded by the healthy retina 1.5 months (M1.5) after the SI. (**E**,**F**) Examples of SD-OCT and fundus images showing the healthy retina of pretreatment (pre). Arrows in C and D indicate the lesion area. Pre: pretreatment. Scale bar: 200 μM.

To overcome the lethal side effect of systemic delivery of retinotoxic reagents or random distribution of the retinal lesion after their intravitreal delivery [15,22], and to facilitate the study of potential therapeutic interventions, subretinal stem cell transplantation in particular, we subretinally injected 100 μL of SNP solutions at three doses (0.05, 0.1, and 0.2 mM) or NS into the posterior pole of the monkey retina next to the fovea under a surgical microscope (Figure 1A). Within 1 h after subretinal injection (SI), SD-OCT images showed that SNP solutions or NS caused a hypo-reflective subretinal bleb, indicating the successful delivery (Figure 1B). The bleb usually disappeared one day after SI, leaving a circular bleb mark, short for bleb area, which was about 8 mm in diameter. During the observation period after administration, with SD-OCT and fundus photographs, the focal, circular lesions with various degrees of severity were clearly noticed and consistently located in the bleb area in all animals including the NS group. The retina outside the lesion in the monkeys was not noticeably affected by the treatment (Figure 1C,D). Therefore, the lesion area at the posterior pole was focal compared to the whole retina. All monkeys were in good health without systemic side effects or death. However, due to the unexpected injury to lens and retinal vessels in operation, complications were observed in a few eyes, including cataracts (2 eyes), vitreous hemorrhage (1), retinal tear and detachment (1), and endophthalmitis (1). 

### 3.2. SNP Caused Acute Retinal Degeneration of Cynomolgus Monkeys in a Dose-Dependent Manner

With the advantage of the noninvasive and time-saving in vivo measurements of retinal layers over histological examination, B-scan and En-face SD-OCT imaging was employed to dynamically evaluate the damage severity and area of retinal structures after the SNP treatment in the monkeys. 

**Figure 2:**
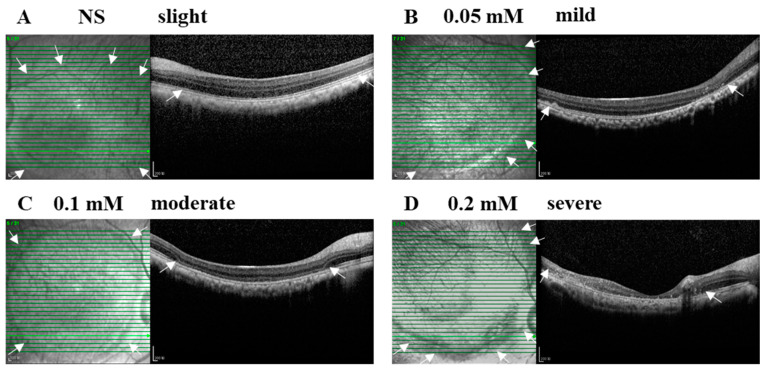
SNP administration induced focal retinal degeneration in monkeys in a dose-dependent manner. (**A–D**) Examples of B-scan (right side) and En-face (left side) SD-OCT images indicate the severity and lesion size of normal saline (NS) and SNP in three doses on day 28 after SI. (**A**) NS caused a slight injury with disorganized photoreceptor segments. (**B**) 0.05 mM led to an uneven mild damage with disorganized photoreceptor segments and partial depletion of the outer nuclear layer at the bleb edge. (**C**) 0.1 mM caused a uniform moderate damage with the depletion of outer retinal layers in the bleb area. (**D**) 0.2 mM caused an uneven severe damage with the whole retina involved in the bleb area. Arrows indicate the edge of the bleb area. Scale bar: 200 μM.

The SI of NS or SNP solutions in three doses led to acute local retinal injuries with various severities from slight, mild, and moderate to severe alterations within 28 days after administration (Figure 2A–D). In the vehicle control group, NS (3/3, 100%) caused a slight injury with disorganized photoreceptor segments. In the 0.05 mM SNP group, the majority of eyes (5/6, 83%) presented an uneven mild injury of the retina with disorganized photoreceptor segments and partial depletion of the outer nuclear layer (ONL) at the bleb edge, and one eye (1/6, 17%) had severe retinal lesions with disruption of most of the retina and choroid. In the 0.1 mM SNP group, 11 out of 14 eyes (11/14, 79%) presented a uniform moderate injury of the retina with the depletion of outer retina layers in the bleb area; 2 out of 14 eyes (2/14, 14%) were mild, and 1 out of 14 eyes (1/14, 7%) was severe. In the 0.2 mM SNP group, four out of five eyes (4/5, 80%) presented an uneven severe injury of the retina with the disruption of the whole retina accompanied by the involvement of the choroid in the bleb area, and one out of five eyes (1/5, 20%) was moderate. In all groups, the retina outside the bleb area remained healthy without evident structural changes.

**Figure 3:**
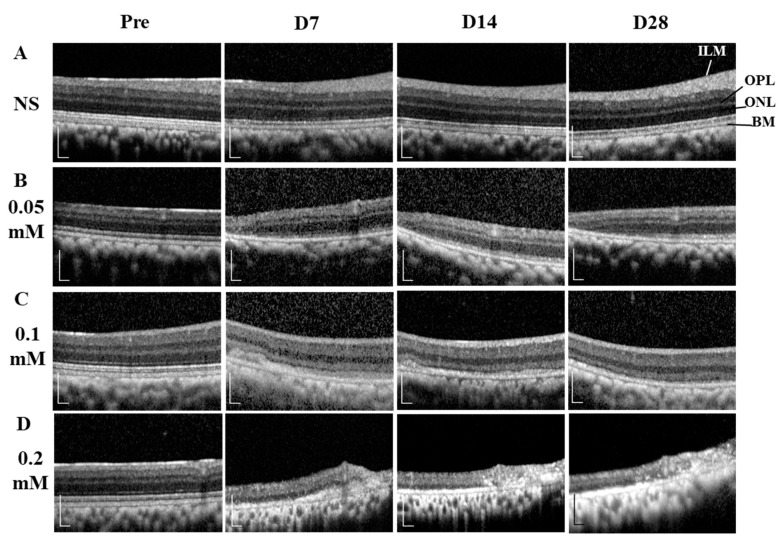
SD-OCT images showing the dynamic changes of retinal structures within 28 days after SNP treatment. (**A**) NS, (**B**) 0.05 mM SNP, (**C**) 0.1 mM SNP, (**D**) 0.2 mM SNP. ILM: internal limiting membrane; OPL: outer plexiform layer; ONL: outer nuclear layer; BM: Bruch’s membrane. Scale bar: 200 μM.

**Figure 4:**
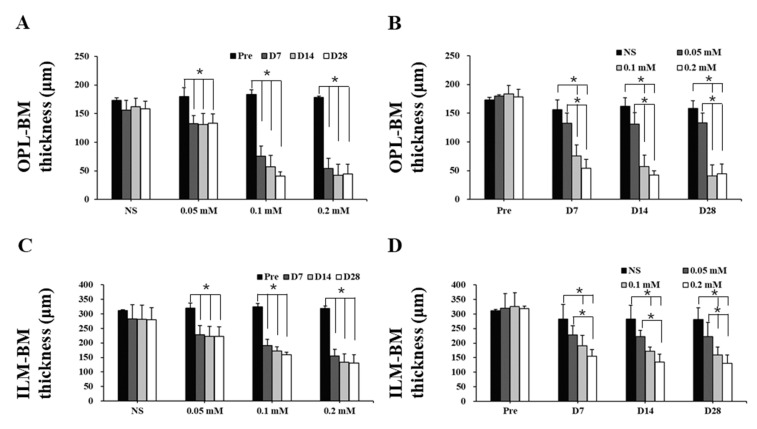
Retinal thickness of monkeys decreased after SNP treatment in both a dose- and time-dependent manner. (**A**,**C**) Compared with pretreatment at each time point, the thickness of the outer retina (**A**) and whole retina (**C**) in the lesion area was not significantly reduced in the NS control group but was significantly reduced in all three doses of the SNP groups. (**B**,**D**) Compared to the NS group, the thickness of the outer retina (**B**) and whole retina (**D**) in the lesion area was not significantly reduced in the 0.05 mM SNP group but was significantly reduced in both the 0.1 and 0.2 mM SNP groups 7 to 28 days after treatment. *: *p* < 0.05.

The time course images of B-scan SD-OCT revealed the pathological changes of cynomolgus monkey retinas over the 28-day observation period (Figure 3 and Figure 4). The disorganization and loss of partial or all outer retinal layers were seen on day 7 and thereafter in all SNP treatment groups, while NS caused only slight disorganization of photoreceptor inner and outer segments. Compared with pretreatment at each time point, the retinal thickness (from the OPL to BM and from the ILM to BM) in the lesion area was not significantly reduced in the NS control group but was significantly reduced in all three doses of the SNP groups (Figure 4A, C). When compared to the NS control group, the retinal thickness (from the OPL to BM and from the ILM to BM) in the lesion area was not significantly reduced in the 0.05 mM SNP group but was significantly reduced in both 0.1 and 0.2 mM SNP groups 7 to 28 days after treatment (Figure 4B, D). Among the three SNP groups, the retinal thickness in the 0.1 and 0.2 mM groups was much thinner than that in the 0.05 mM SNP group. In addition, the retinal thickness in the lesion area of all three SNP groups on days 14 and 28 did not show a more significant reduction compared to that of day 7 (Figure 4A, C). The above results indicated that the subretinal delivery of SNP could induce focal acute retinal damage at the posterior pole of the monkey retina. The severity of retinal damage was dose dependent. 

**Figure 5:**
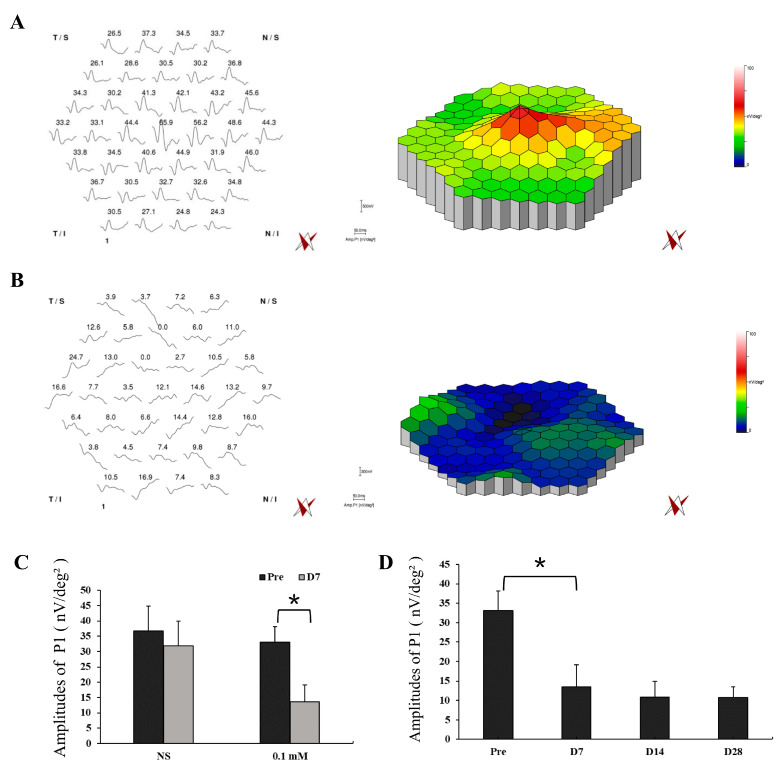
Multifocal electroretinography (MfERG) tests showed a significant reduction in retinal response of the SNP-damaged area. (**A**,**B**) Example of mfERG results in the lesion area of monkey retinas with NS (A) and 0.1 mM SNP (B) treatments on D7 after SI. (**C**) The averaged responses of P1 of pretreatment and on D7 after treatment in the NS group and 0.1 mM SNP group, respectively. (**D**) The averaged responses of P1 in the 0.1 mM SNP group during the 28-day observation period. *: *P* < 0.05.

Functional examination with mfERG was also performed in the lesion area of monkey retinas with NS and 0.1 mM SNP SI with simultaneous infrared fundus monitoring (Figure 5, Figures S1 and S2). NS SI did not cause noticeable changes in the amplitudes of P1 (Amp. P1) between pretreatment (amplitude 36.8 ± 8.1 nV/deg²) and day 7 after treatment (amplitude 31.8 ± 8.2 nV/deg², *P* = 0.49; Figure 5A,C), implying the SI approach itself did not cause evident functional change of the retina. However, 0.1 mM SNP significantly reduced the responses 7 days after treatment (amplitude 13.6 ± 5.6 nV/deg², *P* = 0.01) compared to the pretreatment (amplitude 33.1 ± 5.0 nV/deg², Figure 5B,C). In addition, the reduced retinal responses in the 0.1 mM SNP group did not recover during the 28-day observation period (Figure 5D).

### 3.3. SNP Induced Stable and Long-Lasting Retinal Degeneration in Cynomolgus Monkeys 

**Figure 6:**
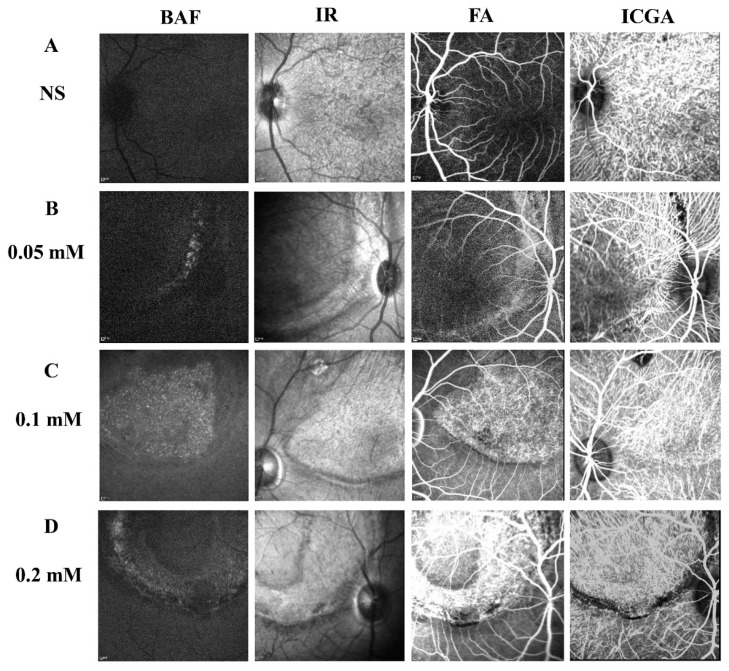
Multimodal images showing the stable and long-lasting retinal degeneration lesions in cynomolgus monkeys. (**A**–**D**) Multimodal imaging of BluePeak autofluorescence (BAF), infrared (IR), fluorescein angiography (FA), and indocyanine green angiography (ICGA) performed in the fifth month for the SNP groups of three doses and the NS control group. The fundus FA images were taken at the arteriovenous phase. The fundus images of ICGA were taken at the early phase.

To determine the long-term effect of SNP SI on the retina, a few monkeys from each group were followed up for more than 5 months after the treatment. Multimodal imaging performed in the fifth month disclosed a consistent focal lesion at the posterior pole of monkey retinas in the SNP groups of three doses (Figure 6). The size and shape of these damaged lesions were close to those described above. Compared to the NS group, both FA and ICGA showed that fluorescence leakage and tissue staining were obvious in the lesion area of the retina in the SNP treatment groups and demonstrated that SNP caused retinal damage in a dose-dependent manner. 

In the seventh month after treatment, mfERG was also performed in the lesion area of cynomolgus monkey retinas with 0.1 mM SNP SI. Compared to the results of the pretreatment and on the 14th day after treatment, the Amp. P1 in the seventh month was significantly reduced (Figure S3), indicating that the SNP administration caused permanent dysfunction of the retina. 

**Figure 7:**
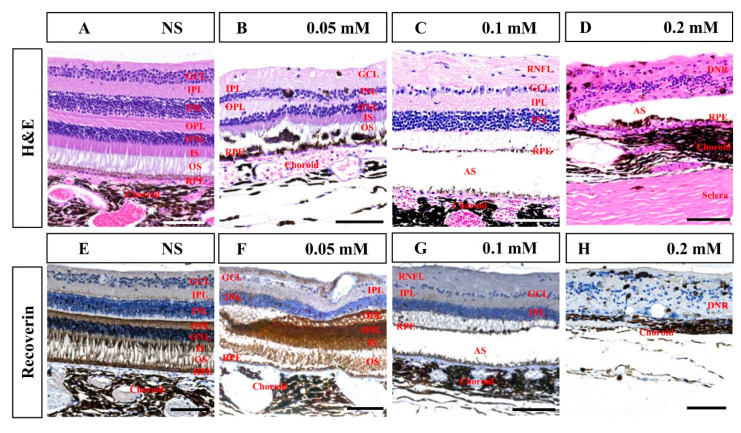
Hematoxylin and eosin (H&E) and immunohistochemistry staining of the retina in monkeys 7 months after SNP SI. (**A**–**D**) H&E staining in the NS group and SNP groups with three different doses. (**E**–**H**) Immunohistochemistry staining showed that recoverin-positive photoreceptor cells showed no remarkable changes in the NS group, were partially lost in the 0.05 mM group, almost entirely disappeared in the 0.1 mM group, and completely disappeared in the 0.2 mM group. GCL: ganglion cell layer; IPL: inner plexiform layer; INL: inner nuclear layer; OPL: outer plexiform layer; ONL: outer nuclear layer; IS: inner segment; OS: outer segment; RPE: retinal pigment epithelium; RNFL: retinal nerve fiber layer; AS: artifact space caused by tissue process; DNR: degenerated neural retina. Scale bar: 100 μM.

Histological examination in the seventh month after SNP treatment confirmed that SNP SI caused focal retinal degeneration of cynomolgus monkeys in a dose-dependent manner (Figure 7), which was consistent with the results of SD-OCT described above. NS caused slight disorganization of the RPE and photoreceptor segments; 0.05 mM SNP caused disorganization of the RPE and photoreceptor segments, and partial loss of ONL; 0.1 mM SNP caused depletion of outer retinal layers, including the RPE, segments, ONL, and OPL; while 0.2 mM SNP destroyed the entire retina and choroid. Some glial scars were observed in degenerated retina in the 0.2 mM SNP group, which did not evidently appear in the 0.05 or 0.1 mM SNP group. Choroidal neovascularization (CNV) and evident inflammatory cells were not clearly observed in the lesion area of the retina from all groups. Compared to the NS control group, immunostaining with the antibody recoverin revealed that photoreceptors were clearly eliminated by 0.1 and 0.2 mM SNP but only partially affected by 0.05 mM SNP in the lesion area of monkey retinas. 

Collectively, the above findings confirmed that the retinal damage induced by SNP SI did not recover in structural and functional levels 7 months after SNP treatment.

## 4. Discussion

In the present study, a single subretinal injection of 100 μL SNP rapidly induced a site-controllable focal retinal degenerative lesion at the posterior pole of the retina in cynomolgus monkeys. The effect of SNP on retinal damage presented dose-dependent changes. Among the three doses (0.05, 0.1, and 0.2 mM) tested, 0.1 mM SNP is the optimal dose that caused focal uniform depletion of the outer neural retina and RPE, leaving the remaining retina healthy. In addition, long-term follow-up demonstrated that the structural and functional injuries of the retina in the lesion area were stable. To our knowledge, this study was the first to fully evaluate the effectiveness and optimal dosage of the subretinal delivery of SNP to develop a focal RD-like monkey model. This focal lesion-site-controllable RD monkey model could provide a valuable platform for developing potential therapeutics and subretinal stem cell therapy for RD patients in particular.

Several approaches, such as intravenous injection, intravitreal injection, and SI, have been applied to deliver retinotoxic reagents to animal eyes to develop RD-like models [15,17,19,22,28,32,33,34,35,36,37]. Each approach has its own advantages and disadvantages. Intravenous injection is a common and easy method of damaging the bilateral retina with large lesion areas in small- and medium-sized animals [17,25,32,33]. However, systemic administration of retinotoxic reagents also affects the general health status of the experimental animals and can even lead to death and tumor formation [15,22,36,37]. For example, Ou et al. reported that a high dose (75 mg/kg) of sodium iodate delivered by intravenous injection, which was needed to induce retinal damage of cynomolgus monkeys, caused acute renal toxicity and one monkey death [22]. Thus, intravenous injection is not applicable for large animals due to expensive experimental cost. In contrast, the intravitreal route had some advantages, such as no severe systemic side effects and allowing loss of the vision in only one eye, leaving a healthy control eye [34]. Nevertheless, previous studies along with our preliminary experiment in monkeys showed that intravitreal administration of retinotoxic reagents led to uncontrollable or random distribution of retinal lesion sites across the whole retina, which was the big issue for subretinal cell replacement therapy [15,23,28,35]. Since this method needs to directly and accurately deliver cells into the subretinal space of the lesion area to replace the lost cells or tissues, lesion sites located at the posterior pole of the retina are highly preferred, facilitating not only the surgical operation but also the follow-up examination with multimodality imaging. The route of SI has been widely used in the field of cell or gene therapy for retinal diseases, leading to targeted delivery [7,38], but is seldom used to deliver retinotoxic reagents for developing RD-like models. In 2016, Shirai et al. firstly applied SI of cobalt chloride to induce RD-like monkey models for transplantation of hPSC-derived retinal sheets [19]. A dose of 40 μL of cobalt chloride at a concentration of 0.25 or 0.30 mg/mL caused a 3-mm-sized circular lesion at the perimacular zone of the retina in four monkey eyes. In the present study, 100 μL of 0.1 mM SNP subretinally delivered to the posterior pole of the monkey retina caused an 8-mm-sized retinal lesion in 14 monkey eyes. The long-term follow-up revealed that the area of the focal lesion did not increase, and the area outside the lesion remained healthy, which is one of the major advantages of SI compared to other routes, although the operation of SI requires a relatively high level of technical skill compared with other delivery methods. 

While the mechanisms of retinal degeneration induced by SNP are still unclear, the role of NO and its related signal pathways are being considered by researchers. SNP is known to release NO, a short-lived free radical gas [26]. Some studies have shown that NO is involved in the pathogenesis of many retinal disorders including RP, AMD, diabetic retinopathy, and ischemia injury [39]. Potent oxidant peroxynitrite formed from NO and oxygen free radicals has a highly toxic effect on neuronal cells, which challenges normal enzymatic function, alters membrane fluidity, and disrupts ionic transport [26,39]. In addition, the retina with an enrichment of polyunsaturated fatty acid is susceptible to free radical toxicity. In retinal degeneration models induced by SNP, findings from many studies including our own show that the primary target of NO toxicity is the photoreceptors, regardless of the delivery route and species, which can be identified in the low dose of the SNP group [27,28,40]. With the increasing dose of SNP, NO toxicity increased accordingly in the whole retina and choroid in the highest dose (0.2 mM) of SNP tested in this study. Peroxynitrite and oxygen free radicals might be major factors in the acute retinal degeneration induced by NO. As a gas of low molecular weight, NO produced by SNP in the subretinal space can pass through the neighboring tissues and cause a toxic effect. 

In the past decade, studies from our laboratory and other groups have demonstrated that hPSCs are able to differentiate into not only the retinal cells but also laminated retinal organoids, including the neural retina and RPE [5,11,12,13,14,41,42]. In 2014, Zhong et al. firstly reported that functional photoreceptors with light sensitivity were also acquired with hPSCs [12]. These great achievements provide huge promise for treating blindness with advanced RDs like RP and AMD with all photoreceptors lost [5,6,7]. The goal of stem cell therapy for RDs is to regenerate or replace the lost photoreceptor cells with hPSC-derived cells to restore the vision function of patients. Late-stage RDs cause photoreceptor degeneration in a large area of the retina, requiring a large number of donor cells for the stem cell therapy. However, it might be currently impractical to restore or replace all the damaged cells of RD patients due to the shortage of donor cells and the limitation of the surgical technique [8]. In humans or nonhuman primates, such as monkeys, the macula, a unique structure responsible for daytime vision and color vision, plays an important role in daily life [16]. Therefore, restoring the macular vision of RD patients is a high priority in clinical settings, which requires a suitable focal RD-like monkey model for preclinical experiments. Although different approaches, such as laser photocoagulation and systemic or intravitreal delivery of retinotoxic reagents, have been attempted to generate an RD model in monkeys, challenges still exist in regard to the lesion size and location and systemic side effect [19,22]. In this study, a focal acute retinal degeneration lesion at the posterior pole of the monkey retina covering the area of the macula was induced within 7 days after the SNP SI and gradually stabilized by day 28. Three doses (0.05, 0.1, and 0.2 mM) of SNP caused various severities of focal retinal injury in monkeys. For subretinal photoreceptor transplantation, 100 μL of 0.1 mM SNP was an optimal dose, which uniformly eradicated the whole outer retinal layers in the local lesion. Our results also suggested that day 28 after subretinal administration of SNP might be the befitting time for retinal cell transplantation.

This study also has some limitations. Compared with intravitreal injection, the operation of subretinal injection requires advanced skills and more training time. Some complications in eyes occurred. SNP-induced retinal degeneration did not exactly represent the pathogenesis of RDs in patients. Individual variation in SNP dose reaction was observed among monkeys. The effective dose window of SNP SI was quite narrow. Further studies will be needed to solve the above issues.

## 5. Conclusions

This study established a rapid and lesion-controllable RD monkey model by subretinal injection of SNP without systemic side effects and found that 0.1 mM was the optimal dose, providing a valuable platform for preclinical evaluation or developing surgical tools for subretinal stem cell therapy for patients with late stage RDs, RP and AMD in particular.

## Supplementary Materials

The following are available online at https://www.mdpi.com/2073-4409/9/11/2468/s1, Figure S1: Example of mfERG from a normal cynomolgus monkey before SNP SI treatment; Figure S2: Infrared fundus images for monitoring mfERG recording in Figure 6; Figure S3: MfERG showed no functional recovery in the SNP-induced retinal degenerative lesion 7 months after 0.1 mM SNP SI.
